# Home isolation capacity after Covid-19 diagnosis in vulnerable communities of two Brazilian cities: TQT Covid-19 Study

**DOI:** 10.11606/s1518-8787.2026060006674

**Published:** 2026-05-01

**Authors:** Audêncio Victor, Fabiane Soares, Diana Zeballos, Thais Aranha Rossi, Joilson Nascimento Paim, Thiago Silva Torres, Debora Castanheira, Valdiléia Gonçalves Veloso, Inês Dourado, Laio Magno

**Affiliations:** I Universidade de São Paulo. Faculdade de Saúde Pública. Programa de Pós-Graduação em Saúde Pública. São Paulo, SP, Brasil; II London School of Hygiene and Tropical Medicine. Faculty of Epidemiology and Population Health. Department of Infectious Disease Epidemiology. London, United Kingdom; III Universidade Federal da Bahia. Instituto de Saúde Coletiva. Salvador, BA, Brasil; IV Universidade Federal da Bahia. Escola Politécnica. Departamento de Engenharia. Salvador, BA, Brasil; V Fundação Oswaldo Cruz. Instituto Nacional de Infectologia Evandro Chagas. Rio de Janeiro, RJ, Brasil; VI Universidade do Estado da Bahia. Departamento de Ciências da Vida. Salvador, BA, Brasil; VII Fundação Oswaldo Cruz. Instituto Gonçalo Moniz. Salvador, BA, Brasil

**Keywords:** Covid-19, Social Isolation, Public Health, Epidemiology, Brazil

## Abstract

**OBJECTIVE:**

To investigate factors associated with self-reported capacity to comply with home isolation after Covid-19 diagnosis in vulnerable communities in two Brazilian cities.

**METHODS:**

Cross-sectional study, with data from a study on the implementation of an intervention based on strategies of testing, isolation, quarantine, and telemonitoring (TQT) of Covid-19 in Primary Health Care in vulnerable neighborhoods (TQT Covid-19 Study). Demographic, socioeconomic, and behavioral data were used to perform descriptive and logistic regression analyses, aiming to evaluate the factors associated with home isolation capacity.

**RESULTS:**

The sample consisted of 324 participants, most of them women (72.5%) and who self-reported to be Black or mixed-race (85.2%). Regarding level of education, 20.1% had up to elementary school degree; 42% had high school degree; and 37.9% had higher education or graduate degree. The density of people per room was high in 57.1% of households. In the multivariate analysis, high household density (≥ 0.5 residents/room) was significantly associated with reduced isolation capacity (OR_a_ = 0.41; 95%CI 0.20–0.82). Other sociodemographic and behavioral variables, including age, sex, race/skin color, level of education, history of Covid-19 infection, access to health services, and preventive behaviors, did not present a statistically significant association.

**CONCLUSION:**

According to the study, housing conditions, especially high household density, can be a determinant for adherence to home isolation. Thus, innovative prevention strategies should combine educational and structural actions that consider the household context of vulnerable families.

## INTRODUCTION

Covid-19, a disease caused by the SARS-CoV-2 coronavirus, emerged as a global crisis in December 2019, leading the World Health Organization (WHO) to declare it a public health emergency of international importance in January 2020^1^. The pandemic stood out as a remarkable event in modern history, representing an unprecedented challenge for public health systems around the world^1,2^. In view of the absence of specific antiviral treatments, and in the expectation for mass vaccination, public health strategies were recommended to contain the spread of the virus^3-5^, including: lockdowns; border closures; and the promotion of practices of hygiene and infection control such as hand hygiene, physical isolation, and the use of masks^3,6^. Conversely, the late adoption of these measures, particularly at a time of the pandemic when vaccines were unavailable, could increase the pressure on health systems for more hospital beds, especially those of intensive care^7,8^.

In this sense, Primary Health Care (PHC) played a crucial role in combating the pandemic through strategies such as testing, clinical screening, social support, care, and telemonitoring of patients until their recovery^4,9,10^. Home isolation emerged as a critical measure to mitigate the spread of the virus. However, its effectiveness was strongly linked to the socioeconomic and housing conditions of the populations^5,11,12^. In countries with great economic inequalities, such as Brazil, vulnerable communities faced unique challenges, which hampered the effective adherence to isolation^13-15^. There are still gaps in the literature; there are no instruments to predict the capacity of individuals and families to comply with isolation during respiratory infections, such as Covid-19, limiting future strategies.

In this context, the TQT Covid-19 Study was carried out in the cities of Salvador/state of Bahia (BA) and Rio de Janeiro/state of Rio de Janeiro (RJ), aiming to implement and expand an intervention based on strategies of Testing, Isolation, Quarantine, and Telemonitoring (TQT) of Covid-19, as well as investigating the factors that hindered and enabled the home isolation capacity after Covid-19 diagnosis to provide subsidies for public policies adapted to urban realities of great vulnerability. Thus, in the present study, we aim to investigate the factors associated with self-reported capacity to comply with home isolation after Covid-19 diagnosis in vulnerable communities in Salvador and Rio de Janeiro. In this sense, with this research, we seek to provide evidence to subsidize public policies more effective and adapted to the realities of vulnerable populations, contributing to future strategies to respond to public health emergencies.

## METHODS

Cross-sectional study with data from the project *Expansão das estratégias de testagem, quarentena, e-health e telemonitoramento para contribuir no enfrentamento da pandemia de Covid-19 no Brasil" (Estudo TQT Covid-19)* ["Expansion of testing, quarantine, e-health and telemonitoring strategies to contribute to coping with the Covid-19 pandemic in Brazil" (TQT Covid-19 Study)], conducted between July 2022 and July 2023^16^. The original project had an epidemiological design with cross-sectional and cohort components.

The participants were seen at PHC services in the health district of Cabula-Beirú, in Salvador (BA), and in the neighborhood of Manguinhos, in Rio de Janeiro (RJ), Brazil. The testing criteria included symptomatic individuals with Covid-19 symptoms three to seven days after their onset. For those who had close contact with a confirmed case, the criterion was the same. For asymptomatic patients, the criterion was five to seven days after contact. The analysis of this article considered all people who had positive results in rapid antigen tests for Covid-19, as shown in Figure.

**Figure:**
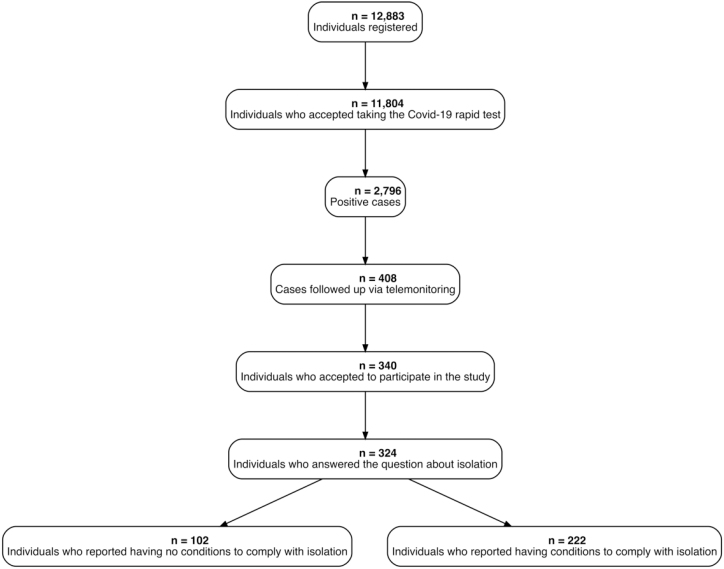
Flowchart of the TQT Covid-19 Study population, 2022–2023.

The choice of PHC centers was guided by formative research, with criteria including:

- PHC services with at least one doctor to follow up patients diagnosed with Covid-19;
- high acceptability of the intervention by health professionals, managers, Community Health Agents (CHA), and local communities;
- PHC services with minimum Internet infrastructure and computer equipment or mobile phone.

The TQT strategy included three subcomponents:

- training and technical support for health professionals;
- recruitment strategies and demand creation;
- testing, isolation, quarantine, and telemonitoring.

Health professionals, municipal managers, and CHA were prepared via virtual and face-to-face training.

### Study Variables

The outcome variable "self-reported capacity to comply with home isolation after Covid-19 diagnosis" was dichotomized in "Individuals who reported having conditions to comply with isolation" (1) and "Individuals who reported having no conditions to comply with isolation" (0), based on the following question: "Can you stay isolated in a room in your house for seven to ten days without having contact with other people?". In addition, socioeconomic, demographic, clinical, and behavioral variables were considered as potential predictors of the outcome, according to previous literature review.

Among the demographic factors, the variable "sex" was categorized into men and women. Age was categorized into three groups: ≤ 25 years, 26 to 49 years, and 50 years or over; while race/skin color was classified into two categories: Black and mixed-race (Black/mixed-race) and white, Asian, and Indigenous (white/Asian/Indigenous). Level of education was classified into three categories: up to elementary school (complete or incomplete), high school (complete or incomplete), and higher education or graduate degree (including undergraduate and graduate courses). Household density was calculated as the number of people per household divided by the number of rooms per household, being categorized into low (< 0.5 residents/room) and high (≥ 0.5 residents/room). The variable "Access to health services" was recategorized into three groups: Brazilian Unified Health System (SUS) (exclusive use of the public system or otherwise), private (exclusive use of health insurance or private care), and both (combined use of public and private services).

The difficulty in accessing the test was evaluated by an objective question, categorizing the answers into "yes" or "no." The history of comorbidities was also considered as a dichotomous variable (yes or no), as well as pregnancy, which identified whether women were pregnant at the time of research. Among the behavioral variables, use of masks and the habit of covering the mouth when coughing were classified into three response categories: "always," "sometimes," and "never."

### Data Analysis

A descriptive analysis of the characteristics of the study population was performed using absolute and relative frequencies for categorical variables. The associations between independent variables and the outcome were evaluated using Pearson's χ^2^ or Fisher's exact tests, when appropriate. Subsequently, binary logistic regression models were adjusted to estimate the association between demographic, socioeconomic, and behavioral factors and the probability of compliance with isolation. In the univariate analysis, all variables were independently tested, and those with p < 0.25 were included in the initial multivariate model.

The final selection of variables was performed by the stepwise backward procedure, based on the Akaike Information Criterion (AIC). Odds ratio (OR) and their respective 95% confidence intervals (95%CI) were estimated. In addition, variables of recognized theoretical relevance and previously associated with differences in adherence to preventive measures during the pandemic, such as age, sex, level of education, and race/skin color, were kept in the adjusted models regardless of the p-value, in order to control potential confounding variables^17-19^. The goodness of fit of the models was evaluated by the Hosmer-Lemeshow test. The significance level adopted was p < 0.05. All analyses were performed using the R software, version 4.1.0 (R Foundation for Statistical Computing, Vienna, Austria).

### Ethical Considerations

This study was conducted in accordance with the guidelines of the Resolution of the National Commission of Ethics in Research (Conep) (No. 466/2012 and 510/2016). The protocol was approved by the WHO Research Ethics Committees (Protocol identification: CERC.0128A and CERC.0128B) and the local Brazilian Institutional Ethics Committees on each place (Protocol identification in Salvador, ISC/UFBA: No. 53844121.4.1001.5030; and, in Rio de Janeiro, INI/Fiocruz: No. 53844121.4.3001.5240, ENSP/Fiocruz: No. 53844121.4.3001.5240, and SMS/RJ: No. 53844121.4.3002.5279).

## RESULTS

### Descriptive Characteristics of the Study Participants

As shown in Table 1, the sample consisted of 324 participants, the majority being women (72.5%). Regarding the age group, 15.1% aged ≤ 25 years; 55.2%, between 16 and 49 years; and 29.6%, 50 years or over. As for race/skin color, 85.2% self-reported to be Black or mixed-race; and 14.8%, white, Asian, or Indigenous. In terms of level of education, 20.1% had up to elementary school; 42% had complete or incomplete high school; and 37.9% had higher education or graduate degree.

**Table 1:** Sociodemographic, behavioral, and housing characteristics and home isolation capacity included in the total analysis. TQT Covid-19 Study.

Variables		Home isolation		p-value^a^
		Individuals who reported having no conditions to comply with isolation n = 102 n (%)	Individuals who reported having conditions to comply with isolation n = 222 n (%)	
Age group				
	≤ 25	16 (33)	32 (67)	0.57
	26–49	53 (30)	126 (70)	
	≥ 50	33 (36)	59 (64)	
Sex				
	Women	64 (30)	149 (70)	0.29
	Men	31 (37)	52 (63)	
Race/skin color				
	Black/mixed-race	84 (32)	181 (68)	0.91
	White/Asian/Indigenous	16 (32)	34 (68)	
Level of education				
	Up to elementary school	16 (36)	29 (64)	0.23
	Complete and incomplete high school	34 (26)	99 (74)	
	Higher education and graduate degree	25 (36)	45 (64)	
Density of people per room				
	Low density (< 0.5)	26 (25)	78 (75)	0.04
	High density (≥ 0.5)	56 (38)	90 (62)	
Pregnancy				
	Yes	2 (33)	4 (67)	1.09
	No	84 (35)	158 (65)	
History of comorbidity				
	No	75 (31)	167 (69)	0.85
	Yes	27 (33)	55 (67)	
History of Covid-19 infection				
	Yes	38 (42)	52 (58)	0.03
	No/do not know	48 (28)	122 (72)	
Access to health services				
	SUS	57 (34)	111 (66)	0.29
	Private	16 (40)	24 (60)	
	Both	13 (25)	39 (75)	
Use of masks				
	Yes	38 (31)	83 (69)	0.89
	No	28 (34)	55 (66)	
	Sometimes	16 (35)	30 (65)	
Cover the mouth or nose when coughing				
	Yes	65 (30)	150 (70)	0.05
	No	14 (54)	12 (46)	
	Sometimes	3 (33)	6 (67)	

a Pearson's χ^2^ test.

The density of people per room was classified as high in 57.1% of households, and low in 42.9%. Most participants reported not being pregnant (96.9%), and 25.9% reported comorbidities. Concerning the history of Covid-19 infection, 27.8% reported having already been infected. Regarding access to health services, 51.9% exclusively used SUS; 12.3%, the private sector; and 35.8%, both systems.

As for preventive measures, 69.1% reported to use masks, 31.5% reported covering the mouth or nose when coughing, and 65.2% reported sanitizing personal objects. In the comparison between groups, we observed a statistically significant association for the density of people per room (p = 0.04), indicating greater difficulty isolating in more crowded households, and for the history of previous infection by Covid-19 (p = 0.03), with a lower proportion of isolation among those previously infected. No statistically significant differences were verified in the other variables.

### Factors Associated with Home Isolation Capacity

In the multivariate analysis, participants living in high-density households (≥ 0.5 people/room) presented a lower chance of reporting conditions to comply with isolation compared to those living in low-density households (OR_a_ = 0.41; 95%CI 0.20–0.82). The goodness of fit of the model, evaluated by the Hosmer-Lemeshow test, indicated an adequate calibration (χ^2^ = 3.04; p = 0.93) (Table 2).

**Table 2:** Crude and adjusted logistic regression models of the association between sociodemographic and behavioral characteristics and home isolation capacity during the Covid-19 pandemic in Brazil.

Variables		Crude model			Model A			Model B (Final)		
		OR	95%CI	p-value	OR	95%CI	p-value	OR_a_	95%CI	p-value
Age group										
	≤ 25	-	-		-	-		-	-	
	26–49	0.63	0.17–2.13	0.53	0.62	0.17–2	0.42	0.64	0.18–2.03	0.50
	≥ 50	0.56	0.14–1.98	0.42	0.54	0.14–1.84	0.33	0.56	0.15–1.87	0.41
Sex										
	Women	-	-		-	-		-	-	
	Men	0.54	0.26–1.15	0.11	0.56	0.27–1.16	0.12	0.50	0.24–1.01	0.05
Race/skin color										
	Black/mixed-race	-	-		-	-		-	-	
	White/Asian/Indigenous	1.28	0.51–3.41	0.60	1.28	0.52–3.37	0.61	1.35	0.55–3.51	0.52
Level of education										
	Up to elementary school	-	-		-	-		-	-	
	Complete and incomplete high school	1.82	0.73–4.46	0.21	1.76	0.72–4.23	0.20	1.88	0.79–4.45	0.15
	Higher education and graduate degree	0.66	0.23–1.82	0.42	0.65	0.25–1.68	0.41	0.66	0.25–1.67	0.42
Density of people per room										
	Low density (< 0.5)	-	-		-	-		-	-	
	High density (≥ 0.5)	0.41	0.20–0.83	0.03	0.43	0.21–0.85	0.02	0.41	0.20–0.82	0.01
History of Covid-19 infection										
	No/do not know	-	-		-	-				
	Yes	0.74	0.39–1.43	0.43	0.75	0.40–1.43	0.43			
Access to health services										
	SUS	-	-							
	Private	0.93	0.37–2.44	0.91						
	Both	1.06	0.43–2.69	0.90						
Use of masks										
	Yes	-	-							
	No	1.02	0.51–2.09	0.92						
	Sometimes	0.88	0.38–2.12	0.83						
Cover the mouth or nose when coughing										
	Yes	-	-		-	-				
	No	0.49	0.16–1.46	0.21	0.48	0.16–1.40	0.22			
	Sometimes	0.68	0.12–4.25	0.71	0.65	0.12–4.01	0.61			

95%CI: 95% confidence interval; OR: odds ratio.

## DISCUSSION

According to our findings, the capacity for home isolation during the Covid-19 pandemic was mainly influenced by housing conditions, especially by the density of people per room. Participants living in more crowded households presented a lower chance of reporting favorable conditions for complying with isolation. This finding highlights the role of structural inequalities in adherence to prevention measures, reinforcing research results that point to precarious housing as a barrier to social isolation in contexts of vulnerability.

This result is consistent with the international literature, according to which high household density can increase the risk of SARS-CoV-2 infection and makes it logistically impossible to separate cases and contacts at home, reducing the effectiveness of isolation even when there is the intention to adhere to the measures. According to evidence of cohorts in the United Kingdom (UK Biobank/REACT; Virus Watch) and analyses in large urban centers (New York), the association between overcrowded/multigenerational housing and higher risk of infection is documented, supporting the structural role of housing conditions in the dynamics of transmission and in the viability of intra-household isolation^20,21^.

In addition to the epidemiological dimension, authors of qualitative studies and mixed methods describe systemic barriers to home isolation, such as lack of space, shared bathrooms, need for face-to-face work, care for dependents, food and financial insecurity, which disproportionately revert to vulnerable populations^22,23^. These factors operate as material constraints to adherence, even among informed and motivated individuals, which helps explain why behavior-centered interventions have limited effect in high-density household contexts.

In our model, preventive behaviors — such as using masks, covering the mouth when coughing, and sanitizing objects — did not remain associated with the outcome after adjustment. According to the literature, there are heterogeneous results on adherence to self-isolation^23^. Authors of population surveys have pointed to suboptimal adherence and determinants such as knowledge, perceived standards, the possibility of working from home, and financial support; researchers have recently highlighted the variability of definition/measurement of "self-isolation," which hinders the comparison between studies^23,24^. Altogether, evidence suggests that, without material and environmental support, the impact of individual behaviors is often superseded by structural limitations.

In the Brazilian context, as per the literature on social determinants of health during the pandemic, socioeconomic vulnerability, informal work, and housing precariousness amplify risks and reduce the ability to comply with non-pharmacological measures^12^. At the same time, PHC arrangements and telemonitoring showed potential to mitigate outcomes in vulnerable areas, but they do not replace structural housing and social protection policies. These findings help to interpret why, in our sample, individual characteristics (sex, age, level of education, race/skin color) and use of health services did not further explain isolation capacity when considering household density^4,17,25^. However, the political context in Brazil during the pandemic, marked by denialist discourses and polarization, may have negatively influenced the adherence to preventive measures among different socioeconomic groups^26^.

Policy-related implications stem directly from these results. According to evidence of quasi-experimental evaluations and policy analyses, provisions of extra-family isolation, for instance, requalified hotels, financial and food support, and compensated leaves, increase the viability of isolation and reduce transmission, especially in overcrowded households^27^. Strategies that combine material support with objective communication tend to maximize adherence. Therefore, purely educational interventions are necessary, but insufficient when barriers are structural.

To fully understand the mechanisms that explain individual vulnerabilities, it is necessary to consider proximal and distal factors. Proximal factors, such as risk perception and access to health resources, have a direct impact on individuals’ capacity and willingness to adhere to isolation. Distal factors, such as socioeconomic and political contexts, shape the environment in which these decisions are made^5,28,29^. In Brazil, the political and economic crisis during the pandemic, aggravated by a denialist administration, exacerbated social inequalities, affecting adherence to public health measures^30,31^.

There are some strengths in this study. The multicentric and diversified sample, from two Brazilian capitals, allowed us to evaluate home isolation capacity in vulnerable urban contexts. The inclusion of sociodemographic, housing, and behavioral variables enabled a comprehensive analysis of the determinants associated with the outcome. The use of multivariate models, with selection by Akaike criteria and adequate calibration, reinforces the consistency of the findings. In addition, we highlight the focus on structural determinants, such as household density, often neglected in studies whose authors prioritize only individual behaviors.

However, limitations should be considered. The cross-sectional design prevents causal inference between exposures and isolation. Self-reported collection may be subject to social desirability bias and classification errors, which tend to attenuate associations. In addition, important socioeconomic variables, such as income and working conditions, were not included due to the high number of missing data, which may have limited the analysis of the structural influence on the isolation capacity. The limited statistical power may have restricted the detection of effects in subgroups. Moreover, recruitment in primary care services and voluntary participation may have introduced selection bias, reducing generalization to other contexts. There is also the possibility of residual confounding by unmeasured variables such as working conditions and support for isolation. Children cannot be isolated on their own, requiring an adult, which can alter the outcome. Furthermore, it was not possible to verify dependence among members of the same household in the sample, potentially introducing uncontrolled correlation.

Future investigations should adopt longitudinal study designs, increase geographic representativeness, and incorporate objective measures, such as mobility indicators and detailed household composition metrics, as well as apply multilevel models to gather contextual effects. In terms of implications, the findings reinforce the need for intersectoral public policies aimed at tackling housing and social determinants, with structural investments for reducing household overcrowding and increasing access to adequate housing conditions.

## CONCLUSIONS

According to our findings, the home isolation capacity during the Covid-19 pandemic was associated with housing conditions, especially the density of people per room. Participants who lived in more crowded households had a lower chance of being able to comply with isolation. This result reinforces the role of structural inequalities in addressing health emergencies, highlighting that, in addition to individual prevention measures, it is essential to consider the housing and socioeconomic context of the most vulnerable populations. Public policies aimed at reducing household overcrowding and increasing access to adequate housing conditions can contribute to greater effectiveness of control strategies in future public health crises.

## Data Availability

The data analyzed in this study are available upon request to the corresponding author.
