# Supplementary material for: Home isolation capacity after Covid-19 diagnosis in vulnerable communities of two Brazilian cities: TQT Covid-19 Study
Source: Rev Saude Publica. 2026 May 1;60(Suppl 1):e6s. doi: 10.11606/s1518-8787.2026060006674 (PMC13138125; doi:10.11606/s1518-8787.2026060006674)
Supplement: Translation

# Translation: Capacidade do isolamento domiciliar após o diagnóstico de Covid-19 em comunidades populares de duas cidades brasileiras: Estudo TQT Covid-19

**Authors:** Audêncio Victor, Fabiane Soares, Diana Zeballos, Thais Aranha Rossi, Joilson Nascimento Paim, Thiago Silva Torres, Debora Castanheira, Valdiléia Gonçalves Veloso, Inês Dourado, Laio Magno

**Affiliations:** I Universidade de São Paulo. Faculdade de Saúde Pública. Programa de Pós-Graduação em Saúde Pública. São Paulo, SP, Brasil; II London School of Hygiene and Tropical Medicine. Faculty of Epidemiology and Population Health. Department of Infectious Disease Epidemiology. London, United Kingdom; III Universidade Federal da Bahia. Instituto de Saúde Coletiva. Salvador, BA, Brasil; IV Universidade Federal da Bahia. Escola Politécnica. Departamento de Engenharia. Salvador, BA, Brasil; V Fundação Oswaldo Cruz. Instituto Nacional de Infectologia Evandro Chagas. Rio de Janeiro, RJ, Brasil; VI Universidade do Estado da Bahia. Departamento de Ciências da Vida. Salvador, BA, Brasil; VII Fundação Oswaldo Cruz. Instituto Gonçalo Moniz. Salvador, BA, Brasil

**Keywords:** Covid-19, Isolamento Social, Saúde Pública, Epidemiologia, Brasil

## Abstract

**OBJETIVO:**

Investigar os fatores associados à capacidade autorreferida de cumprimento do isolamento domiciliar após o diagnóstico de Covid-19 em comunidades vulnerabilizadas de duas cidades brasileiras.

**MÉTODOS:**

Estudo de corte transversal com dados de um estudo de implementação de uma intervenção baseada em estratégias de testagem, isolamento, quarentena e telemonitoramento (TQT) para Covid-19 na Atenção Primária à Saúde em bairros vulnerabilizados (Estudo TQT Covid-19). Foram utilizados dados demográficos, socioeconômicos e comportamentais para realizar análises descritivas e de regressão logística, visando avaliar os fatores associados à capacidade de isolamento domiciliar.

**RESULTADOS:**

A amostra foi composta por 324 participantes, sendo a maioria do sexo feminino (72,5%) e autodeclarada preta ou parda (85,2%). Em relação à escolaridade, 20,1% possuíam até o ensino fundamental; 42%, ensino médio; e 37,9%, ensino superior ou pós-graduação. A densidade de pessoas por cômodo foi alta em 57,1% dos domicílios. Na análise multivariada, a alta densidade domiciliar (≥ 0,5 moradores/cômodo) esteve significativamente associada a menor capacidade de isolamento (OR_a_ = 0,41; IC95% 0,20–0,82). As demais variáveis sociodemográficas e comportamentais, incluindo idade, sexo, raça/cor, escolaridade, histórico de infecção por Covid-19, acesso aos serviços de saúde e comportamentos preventivos, não apresentaram associação estatisticamente significativa.

**CONCLUSÃO:**

O estudo indica que condições habitacionais, especialmente a alta densidade domiciliar, podem ser um determinante para a adesão ao isolamento domiciliar. Assim, estratégias inovadoras de prevenção devem combinar ações educativas e estruturais que considerem o contexto domiciliar de famílias em situação de vulnerabilidade.

## INTRODUÇÃO

A Covid-19, uma doença causada pelo coronavírus SARS-CoV-2, emergiu como uma crise global em dezembro de 2019, levando a Organização Mundial da Saúde (OMS) a declará-la uma emergência de saúde pública de importância internacional em janeiro de 2020^1^. A pandemia destacou-se como um evento marcante na história moderna, representando um desafio sem precedentes para os sistemas de saúde pública ao redor do mundo^1,2^. Diante da ausência de tratamentos antivirais específicos, e na expectativa da vacinação em massa, estratégias de saúde pública foram recomendadas para conter a disseminação do vírus^3-5^, entre elas: *lockdowns*; fechamento de fronteiras; e a promoção de práticas de higiene e controle de infecção, como a higienização das mãos, o isolamento físico e uso de máscaras^3,6^. Por outro lado, a adoção tardia dessas medidas, particularmente em um momento da pandemia em que não havia vacinas disponíveis, poderia aumentar a pressão sobre os sistemas de saúde por mais leitos hospitalares, especialmente aqueles de terapia intensiva^7,8^.

Nesse sentido, a Atenção Primária à Saúde (APS) desempenhou um papel crucial no combate à pandemia por meio de estratégias como testagem, triagem clínica, apoio social, acolhimento e telemonitoramento dos pacientes até sua recuperação^4,9,10^. O isolamento domiciliar emergiu como uma medida crítica para mitigar a propagação do vírus. No entanto, sua efetividade esteve fortemente atrelada às condições socioeconômicas e habitacionais das populações^5,11,12^. Em países com altas desigualdades econômicas como o Brasil, comunidades vulneráveis enfrentaram desafios singulares, o que dificultou a adesão efetiva ao isolamento^13-15^. Ainda há lacunas na literatura; faltam instrumentos para prever a capacidade de indivíduos e famílias cumprirem o isolamento em infecções respiratórias, como a Covid-19, limitando estratégias futuras.

Nesse contexto, o Estudo TQT Covid-19 foi realizado nas cidades de Salvador/BA e Rio de Janeiro/RJ, visando implementar e expandir uma intervenção baseada em estratégias de Testagem, Isolamento, Quarentena e Telemonitoramento (TQT) para Covid-19, bem como investigar os fatores que dificultaram e possibilitaram a capacidade de isolamento domiciliar após o diagnóstico de Covid-19 no intuito de fornecer subsídios para políticas públicas adaptadas às realidades urbanas de alta vulnerabilidade. Assim, o presente estudo tem como objetivo investigar os fatores associados à capacidade autorreferida de cumprimento do isolamento domiciliar após o diagnóstico de Covid-19 em comunidades vulnerabilizadas em Salvador e Rio de Janeiro. Nesse sentido, a pesquisa busca fornecer evidências para subsidiar políticas públicas mais efetivas e adaptadas às realidades das populações vulneráveis, contribuindo para futuras estratégias de resposta às emergências de saúde pública.

## MÉTODOS

Este estudo transversal utilizou dados do projeto "Expansão das estratégias de testagem, quarentena, *e-health* e telemonitoramento para contribuir no enfrentamento da pandemia de Covid-19 no Brasil" (Estudo TQT Covid-19), realizado entre julho de 2022 e julho de 2023^16^. O projeto original teve um desenho epidemiológico com componentes de corte transversal e de coorte.

Os participantes foram atendidos nos serviços de APS no distrito sanitário de Cabula-Beirú, em Salvador, Bahia, e no bairro de Manguinhos, no Rio de Janeiro, Brasil. Os critérios para testagem incluíram indivíduos sintomáticos com sintomas de Covid-19 de 3 a 7 dias após o início. Para aqueles que tiveram contato próximo com um caso confirmado, o critério foi o mesmo. Para assintomáticos, o critério foi de 5 a 7 dias após o contato. A análise deste artigo considerou todas as pessoas que tiveram resultados positivos em testes rápidos de antígeno para Covid-19, conforme mostrado na Figura.

**Figura:** Fluxograma da população de Estudo TQT Covid-19, 2022–2023.

A escolha das unidades de APS foi orientada por pesquisa formativa, com critérios incluindo:

- serviços de APS com pelo menos um médico para acompanhar pacientes diagnosticados com Covid-19;
- alta aceitabilidade da intervenção por profissionais de saúde, gestores, agentes comunitários de saúde (ACS) e comunidades locais;
- serviços de APS com infraestrutura mínima de internet e equipamentos de computador ou telefone móvel.

A estratégia TQT incluiu três subcomponentes:

- treinamento e suporte técnico para profissionais de saúde;
- estratégias de recrutamento e criação de demanda;
- testagem, isolamento, quarentena e telemonitoramento.

Profissionais de saúde, gestores municipais e ACS foram capacitados por meio de treinamentos virtuais e presenciais.

### Variáveis do Estudo

A variável desfecho "capacidade autorreferida de cumprimento do isolamento domiciliar após o diagnóstico de Covid-19" foi dicotomizada em "Indicaram ter condições" (1) e "Indicaram não ter condições" (0), com base na seguinte pergunta: "Você consegue ficar isolado em um cômodo da sua casa de 7 a 10 dias sem ter contato com outras pessoas?". Além disso, variáveis socioeconômicas, demográficas, clínicas e comportamentais foram consideradas como potenciais preditores do desfecho, conforme revisão prévia da literatura.

Entre os fatores demográficos, a variável sexo foi categorizada em masculino e feminino. A idade foi categorizada em três grupos: ≤ 25 anos, 26 a 49 anos, e 50 anos ou mais; enquanto a raça/cor foi classificada em duas categorias: pretos e pardos (preto/pardo) e brancos, amarelos e indígenas (brancos/amarelo/indígenas). O nível de escolaridade foi classificado em três categorias: até o ensino fundamental (completo ou incompleto), ensino médio (completo ou incompleto), e ensino superior ou pós-graduação (incluindo graduação e cursos de pós-graduação). A densidade domiciliar foi calculada como o número de pessoas por domicílio dividido pelo número de cômodos por domicílio, sendo categorizada em baixa (< 0,5 moradores/cômodo) e alta (≥ 0,5 moradores/cômodo). A variável "Acesso aos serviços de saúde" foi recategorizada em três grupos: Sistema Único de Saúde (SUS) (uso exclusivo do sistema público ou outra forma), privado (uso exclusivo de plano de saúde ou atendimento particular) e ambos (uso combinado de serviços públicos e privados).

A dificuldade no acesso ao teste foi avaliada por meio de uma pergunta objetiva, categorizando as respostas em "sim" ou "não". O histórico de comorbidades também foi considerado como uma variável dicotômica (sim ou não), assim como a gestação, que identificou se a mulher estava grávida no momento da pesquisa. Entre as variáveis comportamentais, uso de máscara e hábito de cobrir a boca ao tossir foram classificados em três categorias de resposta: "sempre", "às vezes" e "nunca".

### Análise de Dados

Foi realizada uma análise descritiva das características da população do estudo, utilizando frequências absolutas e relativas para variáveis categóricas. As associações entre variáveis independentes e o desfecho foram avaliadas por meio dos testes χ^2^ de Pearson ou exato de Fisher, quando apropriado. Em seguida, foram ajustados modelos de regressão logística binária para estimar a associação entre fatores demográficos, socioeconômicos e comportamentais e a probabilidade de cumprimento do isolamento. Na análise univariada, todas as variáveis foram testadas de forma independente, e aquelas com p < 0,25 foram incluídas no modelo multivariado inicial.

A seleção final de variáveis foi realizada por procedimento *stepwise backward*, baseado no Critério de Informação de Akaike (AIC, na sigla em inglês). Foram estimados os *odds ratios* (OR) e seus respectivos intervalos de confiança de 95% (IC95%). Além disso, variáveis de reconhecida relevância teórica e previamente associadas a diferenças na adesão a medidas preventivas durante a pandemia, como idade, sexo, escolaridade e raça/cor, foram mantidas nos modelos ajustados independentemente do valor de p, a fim de controlar potenciais variáveis de confusão^17-19^. A bondade de ajuste dos modelos foi avaliada pelo teste de Hosmer-Lemeshow. O nível de significância adotado foi p < 0,05. Todas as análises foram realizadas no software R, versão 4.1.0 (*R Foundation for Statistical Computing*, Viena, Áustria).

### Considerações Éticas

Este estudo foi conduzido em conformidade com as diretrizes da Resolução da Comissão Nacional de Ética em Pesquisa (Conep) (números 466/2012 e 510/2016). O protocolo foi aprovado pelos Comitês de Ética em Pesquisa da OMS (identificação do protocolo: CERC.0128A e CERC.0128B) e pelos Comitês de Ética Institucionais locais brasileiros em cada site (identificação do protocolo em Salvador, ISC/UFBA: n.º 53844121.4.1001.5030; e, no Rio de Janeiro, INI/Fiocruz: n.º 53844121.4.3001.5240, ENSP/Fiocruz: n.º 53844121.4.3001.5240, e SMS/RJ: n.º 53844121.4.3002.5279).

## RESULTADOS

### Características Descritivas dos Participantes do Estudo

Segundo a Tabela 1, a amostra foi composta por 324 participantes, sendo a maioria do sexo feminino (72,5%). Em relação à faixa etária, 15,1% tinham ≤ 25 anos; 55,2%, entre 26 e 49 anos; e 29,6%, 50 anos ou mais. Quanto à raça/cor, 85,2% se autodeclararam pretos ou pardos; e 14,8%, brancos, amarelos ou indígenas. Em termos de escolaridade, 20,1% possuíam até o ensino fundamental; 42%, ensino médio completo ou incompleto; e 37,9%, ensino superior ou pós-graduação.

**Tabela 1:** Características sociodemográficas, comportamentais e de moradia e a capacidade de isolamento domiciliar incluídas na análise total. Estudo TQT Covid-19.

Variáveis		Isolamento domiciliar		Valor p^a^
		Indicaram não ter condições n = 102 n (%)	Indicaram ter condições n = 222 n (%)	
Faixa etária				
	≤ 25	16 (33)	32 (67)	0,57
	26–49	53 (30)	126 (70)	
	≥ 50	33 (36)	59 (64)	
Sexo				
	Feminino	64 (30)	149 (70)	0,29
	Masculino	31 (37)	52 (63)	
Raça/cor				
	Preta/parda	84 (32)	181 (68)	0,91
	Branca/amarela/indígena	16 (32)	34 (68)	
Escolaridade				
	Até o ensino fundamental	16 (36)	29 (64)	0,23
	Ensino médio completo e incompleto	34 (26)	99 (74)	
	Ensino superior e pós-graduação	25 (36)	45 (64)	
Densidade de pessoas por quarto				
	Baixa densidade (< 0,5)	26 (25)	78 (75)	0,04
	Alta densidade (≥ 0,5)	56 (38)	90 (62)	
Gestação				
	Sim	2 (33)	4 (67)	1,09
	Não	84 (35)	158 (65)	
Histórico de comorbidade				
	Não	75 (31)	167 (69)	0,85
	Sim	27 (33)	55 (67)	
Histórico de infecção por Covid-19				
	Sim	38 (42)	52 (58)	0,03
	Não/não sabe	48 (28)	122 (72)	
Acesso ao serviço de saúde				
	SUS	57 (34)	111 (66)	0,29
	Privado	16 (40)	24 (60)	
	Ambos	13 (25)	39 (75)	
Uso de máscara				
	Sim	38 (31)	83 (69)	0,89
	Não	28 (34)	55 (66)	
	Às vezes	16 (35)	30 (65)	
Cobre a boca ou nariz ao tossir				
	Sim	65 (30)	150 (70)	0,05
	Não	14 (54)	12 (46)	
	Às vezes	3 (33)	6 (67)	

a Pearson's χ^2^ test.

A densidade de pessoas por cômodo foi classificada como alta em 57,1% dos domicílios e baixa em 42,9%. A maioria das participantes reportou não estar grávida (96,9%), e 25,9% relataram comorbidades. Em relação ao histórico de infecção por Covid-19, 27,8% informaram já terem sido infectados. Quanto ao acesso aos serviços de saúde, 51,9% utilizavam exclusivamente o SUS; 12,3%, o setor privado; e 35,8%, ambos os sistemas.

No que se refere às medidas preventivas, 69,1% relataram uso de máscara, 31,5% declararam cobrir a boca ou nariz ao tossir, e 65,2% relataram higienizar objetos pessoais. Na comparação entre os grupos, observou-se associação estatisticamente significativa para a densidade de pessoas por cômodo (p = 0,04), indicando maior dificuldade de isolamento em domicílios mais adensados, e para o histórico de infecção prévia por Covid-19 (p = 0,03), com menor proporção de isolamento entre aqueles previamente infectados. As demais variáveis não apresentaram diferenças estatisticamente significativas.

### Fatores Associados à Capacidade de Isolamento Domiciliar

Na análise multivariada, participantes residentes em domicílios com alta densidade (≥ 0,5 pessoas/cômodo) apresentaram menor chance de relatar condições para cumprir o isolamento em comparação àqueles que viviam em domicílios com baixa densidade (OR_a_ = 0,41; IC95% 0,20–0,82). A bondade de ajuste do modelo, avaliada pelo teste de Hosmer-Lemeshow, indicou adequada calibração (χ^2^ = 3,04; p = 0,93) (Tabela 2).

**Tabela 2:** Modelos de regressão logística brutos e ajustados da associação entre as características sociodemográficas e comportamentais e a capacidade de isolamento domiciliar durante a pandemia de Covid-19 no Brasil.

Variáveis		Modelo bruto			Modelo A			Modelo B (Final)		
		OR	IC95%	Valor p	OR	IC95%	Valor p	OR_a_	IC95%	Valor p
Faixa etária										
	≤ 25	-	-		-	-		-	-	
	26–49	0,63	0,17–2,13	0,53	0,62	0,17–2	0,42	0,64	0,18–2,03	0,50
	≥ 50	0,56	0,14–1,98	0,42	0,54	0,14–1,84	0,33	0,56	0,15–1,87	0,41
Sexo										
	Feminino	-	-		-	-		-	-	
	Masculino	0,54	0,26–1,15	0,11	0,56	0,27–1,16	0,12	0,50	0,24–1,01	0,05
Raça/cor										
	Preta/parda	-	-		-	-		-	-	
	Branca/amarela/indígena	1,28	0,51–3,41	0,60	1,28	0,52–3,37	0,61	1,35	0,55–3,51	0,52
Escolaridade										
	Até o ensino fundamental	-	-		-	-		-	-	
	Ensino médio completo e incompleto	1,82	0,73–4,46	0,21	1,76	0,72–4,23	0,20	1,88	0,79–4,45	0,15
	Ensino superior e pós-graduação	0,66	0,23–1,82	0,42	0,65	0,25–1,68	0,41	0,66	0,25–1,67	0,42
Densidade de pessoas por quarto										
	Baixa densidade (< 0,5)	-	-		-	-		-	-	
	Alta densidade (≥ 0,5)	0,41	0,20–0,83	0,03	0,43	0,21–0,85	0,02	0,41	0,20–0,82	0,01
Histórico de infecção por Covid-19										
	Não/não sabe	-	-		-	-				
	Sim	0,74	0,39–1,43	0,43	0,75	0,40–1,43	0,43			
Acesso ao serviço de saúde										
	SUS	-	-							
	Privado	0,93	0,37–2,44	0,91						
	Ambos	1,06	0,43–2,69	0,90						
Uso de máscara										
	Sim	-	-							
	Não	1,02	0,51–2,09	0,92						
	Às vezes	0,88	0,38–2,12	0,83						
Cobre a boca ou nariz ao tossir										
	Sim	-	-		-	-				
	Não	0,49	0,16–1,46	0,21	0,48	0,16–1,40	0,22			
	Às vezes	0,68	0,12–4,25	0,71	0,65	0,12–4,01	0,61			

IC95%: intervalo de confiança de 95%; OR: *odds ratio*.

## DISCUSSÃO

Os achados deste estudo evidenciaram que a capacidade de realizar isolamento domiciliar durante a pandemia de Covid-19 foi influenciada principalmente por condições habitacionais, especialmente pela densidade de pessoas por quarto. Participantes residentes em domicílios mais adensados apresentaram menor chance de relatar condições favoráveis para o cumprimento do isolamento. Este achado evidencia o papel das desigualdades estruturais na adesão às medidas de prevenção, reforçando resultados de pesquisas que apontam a moradia precária como uma barreira ao isolamento social em contextos de vulnerabilidade.

Este resultado é coerente com a literatura internacional, que mostra que a alta densidade domiciliar pode aumentar o risco de infecção por SARS-CoV-2 e torna logisticamente inviável separar casos e contatos em casa, reduzindo a efetividade do isolamento mesmo quando há intenção de aderir às medidas. Evidências de coortes no Reino Unido (*UK Biobank/REACT; Virus Watch*) e análises em grandes centros urbanos (Nova York) documentam a associação entre habitações superlotadas/multigeracionais e maior risco de infecção, sustentando o papel estrutural das condições de moradia na dinâmica de transmissão e na viabilidade do isolamento intradomiciliar^20,21^.

Além da dimensão epidemiológica, estudos qualitativos e de métodos mistos descrevem barreiras sistêmicas ao isolamento em casa, como falta de espaço, banheiros compartilhados, necessidade de trabalho presencial, cuidado de dependentes, insegurança alimentar e financeira, que recaem desproporcionalmente sobre populações vulneráveis^22,23^. Esses fatores operam como constrangimentos materiais à adesão, mesmo entre indivíduos informados e motivados, o que ajuda a explicar por que intervenções centradas apenas no comportamento têm efeito limitado em contextos de alta densidade domiciliar.

No nosso modelo, comportamentos preventivos como uso de máscara, cobrir a boca ao tossir e higienização de objetos não permaneceram associados ao desfecho após ajuste. A literatura mostra resultados heterogêneos sobre adesão ao autoisolamento^23^. Pesquisas populacionais apontaram adesão subótima e determinantes como conhecimento, normas percebidas, possibilidade de trabalhar de casa e suporte financeiro; sínteses recentes destacam a variabilidade de definição/mensuração de "autoisolamento", o que dificulta comparações entre estudos^23,24^. Em conjunto, a evidência sugere que, sem suporte material e ambiental, o impacto de comportamentos individuais é frequentemente suplantado por limitações estruturais.

No contexto brasileiro, a literatura sobre determinantes sociais da saúde durante a pandemia indica que vulnerabilidade socioeconômica, informalidade no trabalho e precariedade habitacional amplificam riscos e reduzem a capacidade de cumprir medidas não farmacológicas^12^. Ao mesmo tempo, arranjos de APS e telemonitoramento mostraram potencial para mitigar desfechos em áreas vulneráveis, mas não substituem políticas estruturais de moradia e proteção social. Esses achados ajudam a interpretar por que, na nossa amostra, características individuais (sexo, idade, escolaridade, raça/cor) e uso de serviços de saúde não explicaram adicionalmente a capacidade de isolamento quando a densidade domiciliar foi considerada^4,17,25^. No entanto, o contexto político no Brasil durante a pandemia, marcado por discursos negacionistas e polarização, pode ter influenciado negativamente a adesão às medidas preventivas entre diferentes grupos socioeconômicos^26^.

Implicações de política decorrem diretamente desses resultados. Evidências de avaliações quase-experimentais e análises de políticas sugerem que ofertas de isolamento extrafamiliar, por exemplo, hotéis requalificados, apoio financeiro e alimentar e licenças remuneradas, aumentam a viabilidade do isolamento e reduzem a transmissão, sobretudo em domicílios superlotados^27^. Estratégias que combinam suporte material com comunicação objetiva tendem a maximizar a adesão. Logo, intervenções puramente educativas são necessárias, porém insuficientes quando as barreiras são estruturais.

Para entender plenamente os mecanismos que explicam as vulnerabilidades individuais, é necessário considerar fatores proximais e distais. Fatores proximais, como percepção de risco e acesso a recursos de saúde, têm impacto direto na capacidade e disposição dos indivíduos em aderir ao isolamento. Já fatores distais, como contextos socioeconômicos e políticos, moldam o ambiente em que essas decisões são tomadas^5,28,29^. No Brasil, a crise política e econômica durante a pandemia, agravada por um governo negacionista, exacerbou desigualdades sociais, afetando a adesão às medidas de saúde pública^30,31^.

Este estudo apresenta algumas fortalezas. A amostra multicêntrica e diversificada, proveniente de duas capitais brasileiras, possibilitou avaliar a capacidade de isolamento domiciliar em contextos urbanos vulneráveis. A inclusão de variáveis sociodemográficas, habitacionais e comportamentais permitiu uma análise abrangente dos determinantes associados ao desfecho. A utilização de modelos multivariados, com seleção pelo critério de Akaike e adequada calibração, reforça a consistência dos achados. Destaca-se, ainda, o foco em determinantes estruturais, como a densidade domiciliar, frequentemente negligenciados em estudos que priorizam apenas comportamentos individuais.

Entretanto, limitações devem ser consideradas. O delineamento transversal impede inferência causal entre exposições e isolamento. A coleta por autorrelato pode estar sujeita a viés de desejabilidade social e a erros de classificação, que tendem a atenuar associações. Além disso, variáveis socioeconômicas importantes, como renda e condições de trabalho, não foram incluídas devido ao alto número de dados ausentes, o que pode ter limitado a análise da influência estrutural sobre a capacidade de isolamento. O poder estatístico limitado pode ter restringido a detecção de efeitos em subgrupos. Além disso, o recrutamento em serviços de atenção primária e a participação voluntária podem ter introduzido viés de seleção, reduzindo a generalização para outros contextos. Também há possibilidade de confusão residual por variáveis não mensuradas, como condições de trabalho e apoio ao isolamento. Crianças não podem fazer isolamento sozinhas, precisando de um adulto, o que pode alterar o desfecho. Além disso, não foi possível verificar dependência entre membros do mesmo domicílio na amostra, potencialmente introduzindo correlação não controlada.

Futuras investigações devem adotar delineamentos longitudinais, ampliar a representatividade geográfica e incorporar medidas objetivas, como indicadores de mobilidade e métricas detalhadas de composição domiciliar, além de aplicar modelos multinível para captar efeitos contextuais. Em termos de implicações, os achados reforçam a necessidade de políticas públicas intersetoriais que enfrentem determinantes habitacionais e sociais, com investimentos estruturais voltados à redução da superlotação domiciliar e à ampliação do acesso a condições adequadas de moradia.

## CONCLUSÃO

Os achados deste estudo evidenciam que a capacidade de isolamento domiciliar durante a pandemia de Covid-19 esteve associada às condições habitacionais, em especial à densidade de pessoas por cômodo. Participantes que residiam em domicílios mais adensados apresentaram menor chance de conseguirem cumprir o isolamento. Esse resultado reforça o papel das desigualdades estruturais no enfrentamento de emergências sanitárias, destacando que, além das medidas individuais de prevenção, é fundamental considerar o contexto habitacional e socioeconômico das populações mais vulneráveis. Políticas públicas voltadas para reduzir a superlotação domiciliar e ampliar o acesso a condições adequadas de moradia podem contribuir para maior efetividade das estratégias de controle em futuras crises de saúde pública.

## Data Availability

Os dados analisados neste estudo estão disponíveis mediante solicitação ao autor correspondente.
